# From Risk to Resilience: A Narrative Synthesis of Protective Factors and Proposed Mechanisms in Bias-Based Bullying Among Youth (2016–2026)

**DOI:** 10.3390/healthcare14213589

**Published:** 2026-10-23

**Authors:** Stephanie F. Dailey, Rosellen Roche, Megan C. Sharkey

**Affiliations:** 1 Counseling Program, School of Education, College of Education and Human Development, George Mason University, Fairfax, VA 22030, USA; msharke@gmu.edu; 2 Department of Family Medicine and Population Health, School of Medicine, Virginia Commonwealth University, Richmond, VA 23298, USA; rosellen.roche@vcuhealth.org

**Keywords:** bias-based bullying, child and adolescent health, conservation of resources theory, ecological systems theory, identity-based bullying, intersectionality, protective factors, resilience

## Abstract

**Highlights:**

**What are the main findings?**

- Protective factors operated across individual, interpersonal, and institutional levels, with relational and institutional resources more frequently associated with protection than individual strengths alone.
- Protection varied according to macro context, intersecting identities and cumulative targeting, and whether school and peer resources were accessible, credible, and usable.

**What are the implications of the main findings?**

- BBB prevention and post-exposure protection should be addressed together through coordinated, identity-responsive supports across ecological systems.
- Healthcare professionals can pair individual support with attention to school climate, adult responsiveness, and coordinated follow-up with schools.

**Abstract:**

Background/Objectives: Bias-based bullying (BBB) is a public health concern associated with psychological distress, suicidality, school disengagement, and other adverse health and developmental outcomes. This review synthesized empirical evidence on protective factors and examined proposed mechanisms and contextual conditions associated with how they operate. Methods: A theory-informed narrative synthesis was conducted using empirical studies published from 2016 through 2026 identified through APA PsycInfo, APA PsycArticles, ERIC, PubMed, Web of Science, and supplementary searches. Fifty-nine studies met the inclusion criteria and were appraised using the Mixed Methods Appraisal Tool. Findings were coded iteratively and interpreted through an integrated ecological and resource-based theoretical framework. Results: Protective factors were identified across individual, interpersonal, and institutional levels. Individual strengths were more often associated with reduced harm than reduced exposure, whereas relational and institutional resources were more frequently associated with protective outcomes. Protective associations varied according to macro climate and policy context, intersecting identities and cumulative targeting, and proximal school and peer activation processes. No single factor was associated with protection for all youth, outcomes, or forms of BBB, and protective associations were often weaker under cumulative exposure. Conclusions: The findings suggest that protection depends on coordinated, accessible, and credible resources across ecological systems. Healthcare professionals should consider identity-based victimization when assessing youth with emotional distress, self-harm risk, disordered eating, sleep problems, or school disengagement, with attention to identity-responsive environments, accessible services, and coordinated follow-up.

## 1. Introduction

Bias-based bullying (BBB) is a distinct form of interpersonal victimization that targets young people because of actual or perceived aspects of identity. Nearly one in five youth in the United States has experienced BBB victimization [1]. In this review, BBB refers to harassment, exclusion, intimidation, or aggression rooted in stigma, bias, or group-based hostility related to race, ethnicity, sexual orientation, gender identity or expression, religion, immigration status, disability, weight or body size, or other marginalized and intersectional identity positions [2,3,4]. Although BBB occurs through peer interactions, it is not reducible to individual conflict between youth. Rather, BBB reflects broader social hierarchies and structural inequities enacted through peer relationships, school climates, and institutional contexts.

Distinguishing BBB from general peer victimization is important because identity-based targeting may carry distinct psychological, academic, and behavioral consequences. When bullying targets characteristics connected to a young person’s identity, family, culture, community, or sense of belonging, the harm may extend beyond the immediate incident. Youth may experience BBB as an attack on who they are or on the groups and communities to which they belong, rather than as an isolated peer conflict. Accordingly, BBB has been associated with elevated risk for depression, anxiety, sadness, hopelessness, suicidal ideation, school avoidance, reduced belonging, and diminished perceptions of school safety [2,5,6,7].

In addition to its distinct harms, BBB often occurs within broader climates of stigma, exclusion, and unequal institutional response [1,2,3,5]. Youth targeted for bias-based reasons may face barriers to disclosure, help-seeking, and support, particularly when school environments do not clearly affirm marginalized identities or respond consistently to identity-based harm. These barriers may be especially consequential for youth who experience multiple or intersecting forms of marginalization, as bias-based victimization can accumulate across identity dimensions. These patterns make BBB relevant to both public health and healthcare practice, because cumulative exposure has been associated with self-injury, suicidal ideation, forgone healthcare, and interpersonal violence [8]. Understanding BBB, therefore, requires attention not only to individual incidents but also to the social, relational, institutional, and structural conditions that shape risk, protection, and recovery.

### 1.1. Integrating Ecological Systems Theory and Conservation of Resources Theory

Ecological Systems Theory (EST) and Conservation of Resources (COR) theory provide complementary frameworks for understanding protective factors in the context of bias-based bullying. EST situates youth development within nested relational, institutional, and structural environments, including families, peers, schools, communities, and broader policy contexts [9]. In this model, youth development is shaped not only by individual characteristics but also by interactions across immediate relationships, schools, communities, and broader social structures. EST is useful for examining BBB because identity-based victimization occurs through peer interactions but is shaped by the larger environments in which those interactions take place.

**Figure 1:**
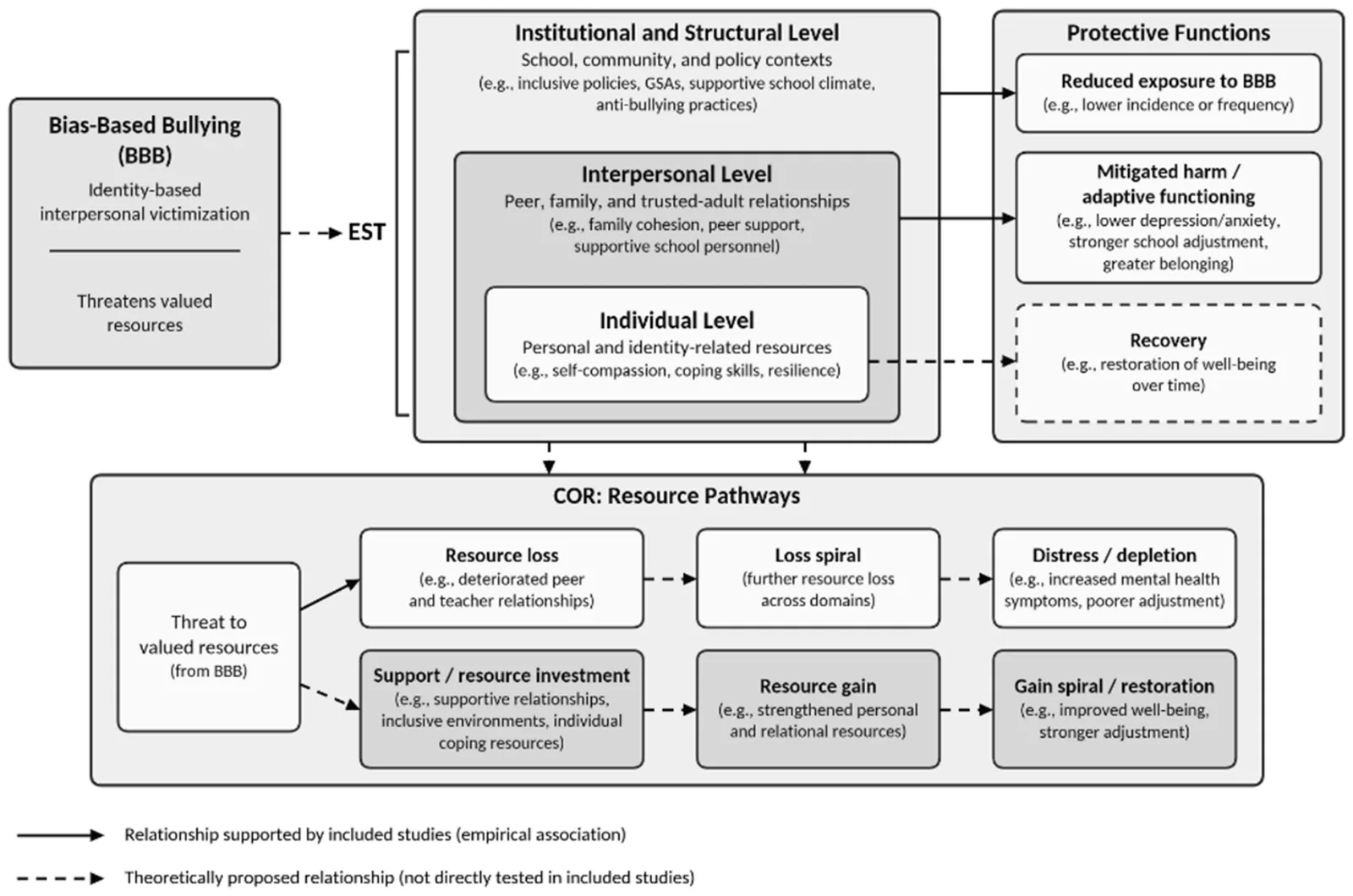
Integrated Ecological and Resource-Based Theoretical Framework. Note. EST = Ecological Systems Theory; COR = Conservation of Resources Theory. This figure presents a proposed interpretive model. Solid arrows indicate relationships directly supported by one or more included studies (associations, not causal effects). Dashed arrows represent theoretically proposed relationships.

COR theory may also help explain why BBB can be especially harmful and why protective factors may not operate equally across contexts. COR theory proposes that stress occurs when valued resources are threatened, lost, or insufficiently restored [10]. Resources refer to valued personal, social, and contextual assets that individuals rely on to maintain well-being, cope with stress, and recover following adversity. In the context of BBB, these resources may include psychological safety, identity affirmation, belonging, trusted relationships, school engagement, and confidence in adult or institutional protection. Together, EST and COR support this review’s focus on both protective factors and the proposed mechanisms and contextual conditions that may shape when, how, and for whom those protective factors are associated with reduced exposure or harm. Figure 1 presents the integrated ecological and resource-based theoretical framework guiding this review.

### 1.2. Aim and Research Questions

The aim of this narrative synthesis is to integrate empirical evidence published between 2016 and 2026 on protective factors associated with bias-based bullying (BBB) among youth. Consistent with the integrated ecological and resource-based framework guiding the review, the synthesis examines both the protective factors associated with reduced exposure to BBB or reduced psychological, social, and educational harm following exposure, and the proposed mechanisms and contextual conditions that may shape when, how, and for whom those protective factors operate. By integrating evidence on both protective factors and the conditions under which they operate, this review informs developmentally responsive, equity-centered approaches to BBB prevention, intervention, and recovery. Accordingly, two research questions guided this review: (1) Which protective factors are associated with reduced BBB exposure or reduced harm following exposure? and (2) What proposed mechanisms and contextual conditions are associated with how and for whom protective factors operate?

## 2. Materials and Methods

### 2.1. Design

This review employed a literature review design with narrative synthesis to identify, integrate, and interpret empirical evidence on protective factors associated with bias-based bullying (BBB) among youth. Consistent with the integrated ecological and resource-based framework guiding the review, mechanisms and contextual conditions were defined as processes, relationships, settings, or broader environments associated with whether protective factors were linked to reduced BBB exposure or reduced harm following exposure. Because few included studies were longitudinal or experimental, mechanisms are described as proposed or inferred pathways and contextual processes unless a study tested them longitudinally or experimentally. Qualitative, quantitative, and mixed-methods studies were eligible for inclusion to capture evidence across individual, interpersonal, institutional, and structural contexts. Narrative synthesis was selected because the included studies varied substantially in research design, participant characteristics, measures of BBB, and conceptualizations of protective factors, making statistical synthesis inappropriate [11]. The review followed an unregistered a priori protocol developed by the review team.

### 2.2. Search Strategy and Data Sources

Between 4 May and 29 July 2026, the review team searched APA PsycInfo, APA PsycArticles, ERIC, PubMed, and Web of Science to identify empirical studies examining protective factors, mechanisms, and contextual conditions associated with BBB among youth. Preliminary scoping searches, beginning in March 2026, informed database selection. The original search strings and date limits were rerun in each database on 29 September 2026, to confirm that they were accurate and reproducible. The PubMed rerun identified one additional eligible study, which was included in the review. The reruns in the other databases identified no new eligible studies. Supplementary searches were conducted during the same initial timeframe using Google Scholar and George Mason University Libraries’ “Mason Catalog and More” discovery search (Primo), with the core search terms. Results were screened in order of relevance, and two additional records were retained from each source. The number of results screened was not recorded. One additional article was identified from the reference lists of included articles.

Search strategies combined keywords and Boolean operators related to bias-based bullying and protective, promotive, or contextual factors. Core search terms included “bias-based bullying” and its common misspelling “biased-based bullying,” as well as expanded combinations of terms related to identity-based bullying, victimization, harassment, bias, prejudice, discrimination, protective factors, resilience, promotive factors, school climate, school belonging, peer support, connectedness, and bystander behavior. The review team adapted the search syntax for each database while maintaining conceptual consistency across searches. Supplemental Table S2 provides the complete database-specific search strategies, search dates, and applied limits.

The search was limited to peer-reviewed, full-text empirical studies published in English. The English-language restriction reflected the lack of resources for translation and verification, and grey literature was excluded to maintain a consistent evidentiary base. APA PsycInfo and APA PsycArticles were searched together through EBSCOhost. Full-text searching was enabled so that articles in which BBB terminology appeared only in the body text, rather than in the title or abstract, were also retrieved, and overlapping records between the two databases were removed during deduplication. CINAHL and Embase were not searched because their coverage of journals relevant to school-based youth bullying was expected to overlap substantially with PubMed and Web of Science. Records were managed in Zotero and Excel, with duplicates reconciled using title, author, publication year, DOI, and full-text comparison before screening.

### 2.3. Inclusion and Exclusion Criteria

Studies were eligible for inclusion in the narrative synthesis if they were published in English between 2016 and 2026, focused on children or adolescents (defined as individuals up to 18 years of age), and examined protective factors, mechanisms, or contextual conditions associated with BBB. Retrospective studies of young adults were eligible only when they reported BBB experiences that occurred during childhood or adolescence. Eligible studies included qualitative, quantitative, and mixed-methods empirical studies that contributed evidence on individual, interpersonal, institutional, or structural factors that may prevent, buffer, or mitigate BBB exposure or its psychological, behavioral, social, educational, or health-related consequences. Studies were eligible if they examined bullying related to actual or perceived identity characteristics, including race, ethnicity, religion, disability, sexual orientation, gender identity or expression, immigration status, nationality, weight or body size, or other marginalized or intersectional identities. Studies were excluded if they focused exclusively on general bullying without addressing bias-based or identity-based bullying, examined discrimination or prejudice in the absence of bullying, focused on adult populations without youth-specific findings, or were published in languages other than English. Editorials, books, book chapters, book reviews, conference abstracts, conceptual articles, literature reviews, and other nonempirical publications were excluded from the final synthesis, although some were retained in the broader corpus for background, construct clarification, or theoretical framing.

### 2.4. Screening, Selection, and Data Extraction

**Figure 2:**
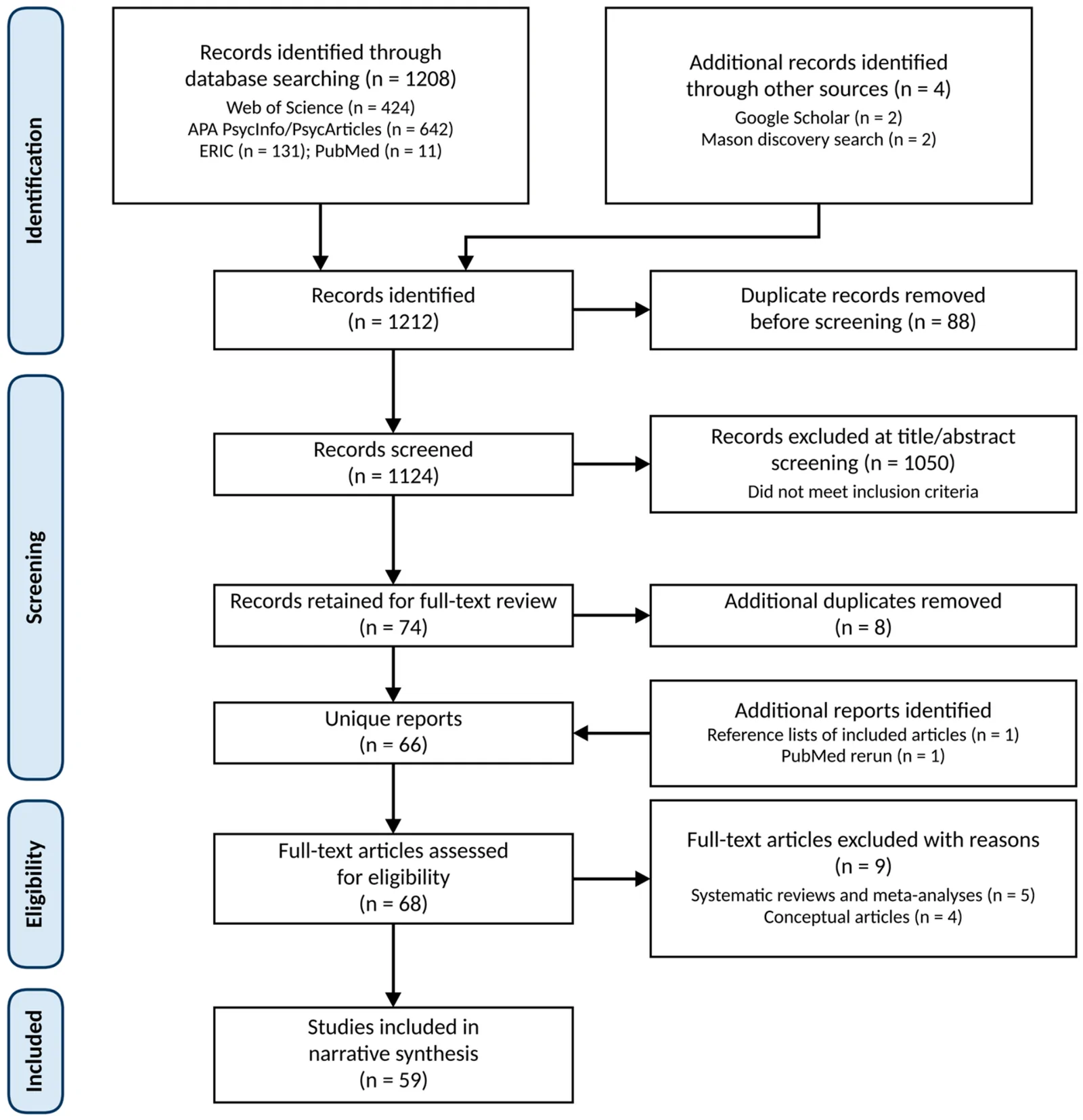
PRISMA flow diagram of study identification, screening, and inclusion.

Following completion of the database and supplementary searches, records were organized in Zotero and exported to a master Excel workbook for screening, selection, and extraction. Zotero was used to manage references, identify potential duplicates, and support retrieval of full-text articles, while the Excel workbook served as the primary screening, extraction, and coding matrix. Figure 2 summarizes the identification, screening, eligibility assessment, and inclusion of studies. Two reviewers screened all records and assessed full texts together, and a third reviewer was consulted to reach consensus on uncertain decisions. Two coders then independently assigned each study’s primary factor type (protective factors, mechanisms, or both). Agreement was 86.2% (κ = 0.79) across the 58 studies coded by both, and coding disagreements were resolved through consensus discussion. The study identified through the updated PubMed search was added after dual coding was completed and was not included in the agreement calculation.

Methodological quality was appraised using the Mixed Methods Appraisal Tool (MMAT, version 2018) [12], which provides design-specific criteria for qualitative, randomized, nonrandomized, and descriptive studies. Before rating, the team agreed on decision rules for representativeness, completeness of outcome data, confounding, and measurement, and applied them uniformly across studies. The confounding criterion was rated as met when studies adjusted for relevant confounders, stratified analyses by key groups, or used a comparison-group design. Decision-tree and latent class analyses that included demographic characteristics without identifying or adjusting for confounders of the associations under study were rated as not meeting this criterion. MMAT ratings were used to appraise the quality of the evidence rather than to determine eligibility, and no study was excluded based on methodological quality.

Data from included studies were extracted into the matrix over three iterative rounds of extraction and coding. The first round captured descriptive study information, including bibliographic details, study purpose, sample characteristics, longitudinal nature and timeframe, type of bias-based bullying, outcomes, risk factors, protective or buffering factors, and key findings. Coding decisions were discussed iteratively among the review team and reconciled at the end of each round.

### 2.5. Data Synthesis Approach

This review integrated findings using an inductive, theory-informed narrative synthesis approach. EST informed a priori categories for organizing factors across individual, interpersonal, institutional, and broader contextual levels. Within these categories, successive rounds of coding inductively identified and refined protective factors, mechanisms, and contextual conditions. COR theory was applied during interpretation rather than to predetermine codes. Concepts such as resource loss, resource caravans, resource passageways, and activation conditions were used to interpret patterns across studies.

Synthesis involved iterative extraction, coding, comparison, and refinement of analytic categories, with the extraction matrix updated alongside the synthesis (see Supplemental Data S1). Protective findings were coded separately for reduced BBB exposure and reduced harm following exposure. Recovery over time was rarely examined and is treated as a theoretically proposed function (see Figure 1). The matrix also recorded each study’s design and whether findings reflected cross-sectional associations, tested moderation or mediation, longitudinal prediction, or experimental or quasi-experimental comparisons, distinguishing mechanisms tested in primary studies from those proposed by study authors or inferred through the review’s framework.

The synthesis also accounted for studies drawing on shared datasets, survey programs, or overlapping samples. Of the 59 included studies, 36 (61.0%) were part of a dataset or sample group represented in more than one study. The primary recurring data sources were the California Healthy Kids Survey (CHKS; 11 studies), Minnesota Student Survey (MSS; 8 studies), Dane County Youth Assessment (5 studies), LGBTQ National Teen Survey (4 studies), National Crime Victimization Survey School Crime Supplement (NCVS-SCS; 4 studies), AmeriSpeak/Peer Experiences Project (2 studies), a shared middle-school sample (2 studies), and a European SGM school survey (2 studies). Because some studies drew on more than one recurring data source, these counts are not mutually exclusive. When assessing the consistency of findings, multiple studies that used the same data source were not considered independent evidence of replication. In this synthesis, consistency referred to the recurrence and direction of associations across studies rather than pooled effect size or comparative efficacy.

## 3. Results

### 3.1. Overview of Included Studies

The final narrative synthesis included 59 empirical studies published between 2016 and 2026. The included studies varied in design, data source, sample size, identity focus, and conceptualization of BBB. Most studies used a quantitative cross-sectional design (*n* = 42), with qualitative studies (*n* = 5), repeated cross-sectional survey analyses (*n* = 4), quasi-experimental studies (*n* = 4), longitudinal panel studies (*n* = 2), and experimental studies involving randomized conditions (*n* = 2) also represented. Samples included middle school, high school, and adolescent survey populations, as well as educator or community informant samples and a small number of retrospective or young-adult samples when findings were directly relevant to youth BBB experiences. Several studies drew on large-scale school or population-based datasets, whereas qualitative, experimental, and intervention studies generally used smaller purposive or program-based samples. Distinctive designs included a statewide surveillance study that used expanded sexual and gender identity categories [13] and a nationally representative retrospective comparison of peer and sibling victimization [14]. Most studies were conducted in the United States (*n* = 47). Others were conducted in Canada (*n* = 2) and in Australia, Germany, Italy, Portugal, Saudi Arabia, Spain, and the United Kingdom (one study each). Three studies used multinational European samples. Supplemental Table S1 summarizes the design, sample, data source, and primary analytic focus of each included study.

The included studies addressed BBB related to sexual orientation, gender identity or expression, race, ethnicity, immigration or national origin, religion, disability, weight or body size, and multiple or intersecting identity positions. Studies also differed in their primary analytic contribution. Thirteen studies primarily examined protective factors, 18 primarily examined mechanisms or contextual conditions, and 28 addressed both (see Table 1). Across the data extraction matrix, the review team coded protective factors most often at the institutional level, followed by interpersonal and individual levels. Mechanism-focused evidence addressed macro climate and policy context, intersectionality and cumulative targeting, and proximal school and peer activation processes (see Table 2). Because studies could contribute to more than one analytic category, these coding patterns are descriptive rather than mutually exclusive.

Methodological quality ratings are presented by criterion in Supplemental Table S3. All five qualitative studies and the single descriptive study met all MMAT criteria. Of the two randomized trials, one met two of five criteria and the other met one. Neither reported its randomization procedure in enough detail to judge. One trial did not report baseline comparability or blinding, and the other reported baseline differences between groups and used unblinded self-reported outcomes. Among the 51 nonrandomized studies, all used appropriate measures, 49 used samples representative of their target populations, 36 accounted for confounders, and 48 reported exposures consistent with the study design. Completeness of outcome data was the least consistently reported criterion, with 13 studies reporting insufficient information.

The findings are presented in relation to the two research questions. The first focused on protective factors associated with reduced BBB exposure or reduced harm following exposure, and the second addressed mechanisms and contextual conditions that shaped how and for whom those factors operated.

### 3.2. Protective Factors Associated with Reduced Exposure or Harm

Using Garmezy’s [15] definition, the review team classified a factor as protective when a study associated it with lower BBB exposure, reduced psychosocial harm following victimization, or more adaptive responses among youth exposed to BBB. Protective factors were organized into the individual, interpersonal, and institutional categories informed by EST, and recurring subcategories within each category were identified inductively through cross-study comparison.

#### 3.2.1. Individual Protective Factors

Individual protective factors constituted the smallest evidence category. Across these studies, individual-level factors clustered around two subcategories, self-awareness and prosocial behavior. These factors were more consistently associated with reduced harm, adaptive functioning, or lower perpetration-related involvement than with reduced exposure to BBB.

**Self-Awareness.** Self-awareness-related factors included self-compassion, self-esteem, LGBTQ or queer identity pride, and broader psychological strengths. Studies linked these factors to coping and adaptive functioning following BBB exposure, but did not show that individual strengths alone offset identity-based victimization. Vigna et al. [16] found that self-compassion attenuated the association between BBB and symptoms of anxiety and depression among sexual and gender minority youth, but this protective association weakened as bullying exposure increased. Similarly, O’Malley et al. [17] found that covitality was associated with lower odds of suicidal thoughts among sexual and gender minority youth but did not significantly buffer the association between homophobic or gender-based bullying and suicidal thoughts. Across studies, these resources were more consistently associated with coping than with reduced BBB exposure.

**Prosocial Behavior.** Evidence on prosocial behavior and related social–emotional competencies centered on perpetration-related involvement rather than victimization. Zych and Llorent [18] found that higher social awareness and prosocial behavior were associated with lower odds of bias-based cyberperpetration and cyberbully-victim involvement among adolescents but not with lower odds of bias-based cybervictimization. Prosocial behavior was also reflected in active bystander responses. Hitti et al. [19] found that adolescents with lower discriminatory tendencies reported greater desire for social contact with immigrant-origin peers, which in turn was associated with stronger intentions to intervene when those peers experienced bias-based bullying. Khalil et al. [20] provided related intervention evidence through a bullying and prejudice prevention program that targeted knowledge, attitudes, empathy, and willingness to intervene. Findings were mixed, with stronger effects for knowledge and attitudes than for empathy or intervention skills. The trial met one of five MMAT criteria, and the authors attributed improvements in the control group partly to the Hawthorne effect, so these findings should be interpreted with caution. Across these studies, prosocial competencies and intergroup attitudes were associated with lower perpetration-related involvement and greater willingness to help targeted peers rather than with lower BBB victimization.

#### 3.2.2. Interpersonal Protective Factors

Interpersonal protective factors captured supportive relationships, social connections, and peer or adult responses within youths’ immediate social environments. Across the included studies, these factors clustered around relatedness, family cohesion and acceptance, and bystander intervention and allyship.

**Relatedness.** Relatedness emerged as a protective resource associated with school connection, adaptive functioning, and reduced psychosocial harm following BBB exposure. Studies in this area emphasized peer support, school belonging, supportive adult relationships, and affirming relational spaces. Parodi et al. [1] found that peer support was associated with lower initial BBB victimization in single-predictor models, although this association attenuated after accounting for other protective factors. School belonging and peer support were associated with lower depression and anxiety and higher academic functioning after accounting for direct and witnessed bias-based harassment, although adult support at school was more consistently associated with academic functioning than with internalizing outcomes [21]. Other studies identified school and community membership, caring adults, peer solidarity, LGBTQIA+ clubs, and inclusive classrooms as relational resources for sexual and gender minority youth, particularly transgender and gender nonconforming youth [16,22,23]. Although some findings linked relational resources to lower BBB exposure, the evidence more consistently associated them with coping, psychological well-being, perceived safety, and school connection. In contrast, Ogg et al. [24] found that positive teacher–student and student–student relationships did not predict lower subsequent exposure to biased language.

**Family Cohesion and Acceptance.** Family-related evidence was more limited than peer and school-based relational evidence, but available studies suggested that family resources may buffer harm and, in some cases, relate to reduced exposure. Shah et al. [25] found that family cohesion buffered associations between immigrant-status-based bullying and internalizing problems, with protective patterns differing by immigrant status and outcome. Parodi et al. [1] found that family support was associated with lower initial BBB victimization in single-predictor models, although this association attenuated after accounting for other protective factors, particularly school climate. Qualitative findings further suggested that family acceptance may form part of broader supportive networks for transgender and gender-nonconforming youth [23].

**Bystander Intervention and Allyship.** Bystander intervention and allyship reflected an active form of interpersonal protection through which peers could interrupt BBB, support targeted youth, or reinforce inclusive norms. Experimental and vignette-based studies linked common inclusive identity framing, intergroup contact, theory of mind, and positive peer norms with greater intentions to defend targeted youth or seek help [26,27]. Hitti et al. [19] found that inclusive peer norms and greater desire for social contact were associated with adolescents’ willingness to intervene when immigrant-origin peers experienced bias-based bullying, with desire for social contact statistically accounting for this association in a cross-sectional mediation model. Other evidence showed that bystander intentions and reported intervention varied across students’ social identities and experiences [28], while youth accounts identified direct defense and peer support as possible responses to BBB [29]. Collectively, these studies identified bystander intervention and allyship as potential interpersonal protections, although most assessed intentions or self-reported responses rather than reductions in BBB exposure or harm.

#### 3.2.3. Institutional Protective Factors

Institutional protective factors included structures, practices, and resources at the school, district, and policy levels that shaped whether youth experienced schools as safe, equitable, and responsive to BBB. This category contained the largest body of evidence on protective factors. Studies clustered around four areas. These were gender–sexuality alliance (GSA) presence, school personnel support and institutional practices, school climate and safety, and inclusive policies and resources.

**GSA Presence.** GSA presence was associated with lower or less steeply increasing BBB exposure across three independent datasets, particularly for sexual and gender minority youth. Gower et al. [30], Lessard et al. [4], and Lessard et al. [31] associated GSA presence with lower exposure to several forms of BBB. The two Lessard studies, which drew on the same LGBTQ National Teen Survey dataset, further found that reduced BBB exposure statistically accounted for associations between GSA presence and lower stress, fewer sleep problems, lower depression, fewer unhealthy weight-control behaviors, and stronger school adjustment. Hatzenbuehler et al. [32] found that schools with GSAs experienced smaller increases in homophobic bullying during the period surrounding California’s Proposition 8 vote. However, the protective reach of GSAs was not uniform. Evidence was weaker or absent for some forms of BBB, including race- or ethnicity-based bullying, and effects varied by student subgroup [30,31].

**School Personnel Support and Institutional Practices.** Evidence concerning school personnel support and institutional practices identified caring school personnel, supportive practices, and LGBTQ-specific resources as institutional protective factors. Day et al. [33] found that supportive practices were associated with lower homophobic bullying and stronger school connectedness, whereas punitive practices were associated with neither outcome. McCauley et al. [34] found that school personnel support buffered depressive symptoms among youth reporting high levels of identity-based harassment, whereas the combination of personnel support and LGBTQ-specific resources was most protective at lower harassment levels. These patterns did not extend to anxiety. Gower et al. [35] linked teacher caring more closely to academic outcomes than to safety-related absences, which were more strongly differentiated by BBB exposure. Overall, supportive personnel and practices showed clearer protective associations than punitive approaches, although their effects varied across outcomes and levels of harassment.

**School Climate, Safety, Fairness, and Equity.** Studies identified school climate, safety, fairness, and equity as distinct institutional protective factors. Parodi et al. [1] found that positive school climate was associated with lower baseline cumulative BBB exposure, although it did not predict change over time. Sherwood et al. [36] linked students’ perceptions of school safety and peer support, but not school-level averages or teacher support, with lower BBB exposure. Fu et al. [37] found that school equity buffered adjustment difficulties following race-based bullying, although effects varied across racial and ethnic groups and outcomes. Mulvey et al. [38] found that the benefits of social support did not extend to youth experiencing BBB, and school safety did not help youth facing the most compounded targeting. Across studies, these institutional conditions were associated with better outcomes for some groups, but no factor operated uniformly across exposure profiles.

**Inclusive Policies, Practices, and Resources.** Studies distinguished broad policy indicators from concrete school practices and resources. Kidwai and Smith [39] found no significant association between policy comprehensiveness and reported bullying, while Parodi et al. [1] found that enumerated state antibullying laws predicted lower initial BBB victimization only before school climate entered the model. Teacher training, inclusive curricula, diversity education, LGBTQ-inclusive resources, and structured peer interventions were associated with lower BBB exposure or harm in several studies [3,40,41,42,43]. These effects still varied across student groups and implementation conditions. For example, diversity education produced different associations for boys of color and White boys, and in a quasi-experimental evaluation, peer-led prevention was associated with reduced ethnic victimization only when peer educators included youth with immigrant backgrounds [3,43]. Overall, concrete practices and resources were more frequently associated with protection than broad policy indicators alone.

### 3.3. Proposed Mechanisms and Contextual Conditions Associated with Protective Factors

The first research question identified individual, interpersonal, and institutional resources associated with lower BBB exposure or harm. The second research question examined why these resources did not operate uniformly across youth, contexts, and outcomes. Through iterative comparison, the review team organized the evidence into three analytic categories, which were macro climate and policy context, intersectionality and cumulative targeting, and proximal school and peer activation processes. These categories were not mutually exclusive because broader context, identity-based exposure, and immediate school and peer responses often operated together. Because most of this evidence was cross-sectional, these mechanisms are described as proposed or inferred pathways and contextual processes unless a study tested them longitudinally or experimentally.

#### 3.3.1. Macro Climate and Policy Context

Across 12 articles, macro climate and policy context were associated with BBB risk and with the broader institutional conditions in which school protections operated. Political events, legal protections, community context, and broader identity-related climates were associated with BBB prevalence and the legitimacy of school responses.

Policy-focused studies found stronger associations for implementation and enforceable remedies than for broad legal protections alone. Parodi et al. [1] found that enumerated state antibullying laws predicted lower initial BBB victimization only before school climate entered the model. Ioverno [40] associated teacher training and inclusive curricula more strongly with lower bias-based school violence than antidiscrimination laws or government support. In a quasi-experimental analysis, Hatzenbuehler et al. [44] found that litigation resulting in a remedy favorable to students was associated with reduced homophobic bullying both within the schools involved and elsewhere in the same districts.

Climate-focused studies found that broader social conditions did not affect all forms of BBB uniformly. Hatzenbuehler et al. [32], drawing from the California Healthy Kids Survey (CHKS), found that homophobic bullying increased during the period surrounding California’s Proposition 8 vote, although schools with GSAs experienced smaller increases. Gill and Govier [45], utilizing the same CHKS dataset, found mixed and largely null changes following the 2016 election cycle, with patterns varying by BBB type, time period, school level, and county voting context. Gower et al. [30] linked community LGBTQ supportiveness with lower bullying based on sexual orientation and weight or appearance among youth assigned female at birth, but found no comparable protection for boys. Sherwood et al. [36] found no direct association between national sexual and gender minority inclusivity and BBB. National climate mattered only in combination with school outness, school safety, and peer support.

Qualitative studies showed how broader ideological and policy environments shaped institutional recognition of BBB. Newman et al. [46], Shank and Troshynski [23], and Ramirez et al. [47] showed how religious ideologies, school board debates, and broad antibullying mandates communicated norms about which identities and experiences schools recognized and protected. Across studies, macro conditions were associated with the distribution of BBB risk and with the institutional environment in which school-level protections operated.

#### 3.3.2. Intersectionality and Cumulative Targeting

Seventeen studies were coded to intersectionality and cumulative targeting, four of which directly quantified cumulative BBB exposure. Studies varied in how intersectionality was operationalized. Some included multiple identity characteristics or examined subgroup differences or interactions among identities, whereas others used person-centered approaches, including latent-class, profile, classification-tree, and decision-tree analyses, to examine combinations of social positions simultaneously. A smaller subset explicitly framed their analyses through an intersectional lens. Thus, the inclusion of multiple identity variables was not treated as equivalent to an explicitly intersectional analytic approach.

Across this evidence, intersecting identities shaped who experienced BBB, where harm concentrated, and whether protective factors remained available or sufficient. Fish et al. [48] reported that age-related increases in BBB and depressive symptoms varied by the interaction of sex, sexual orientation, and gender identity. Galán et al. [8] similarly reported that youth holding multiple stigmatized identities, including racial or ethnic minority and sexual minority identities, experienced the highest rates of both perpetrating and being targeted by identity-based bullying.

Several studies used classification trees, latent classes, or profile analyses to examine intersecting identities simultaneously, drawing on data from the Minnesota Student Survey (MSS) and the CHKS. Eisenberg et al. [49] found the highest emotional distress among youth exposed to BBB who held both sexual and gender minority identities, compared with youth who held the same identities without BBB exposure. Lawrence et al. [50] identified BBB as a defining characteristic of sexual risk profiles among cisgender girls who held additional marginalized positions, most often physical disability or a lesbian or gay identity. Among cisgender girls with physical disabilities, 20% of those exposed to BBB reported alcohol or drug use before sex, compared with 12% of those without recent BBB exposure. Meng et al. [51] found that BBB further differentiated suicidal ideation among youth who otherwise shared similar combinations of race, sex assigned at birth, gender identity, and sexual orientation. Across two state datasets, Gower et al. [52] found the highest BBB prevalence among transgender, gender diverse, and gender questioning youth who held additional marginalized social positions.

Related person-centered studies extended these patterns beyond mental health, drawing on the Dane County Youth Assessment and the MSS. Parchem et al. [53] identified a latent class characterized by BBB as the primary reported barrier to organized physical activity. Across matched comparisons that could be tested, Parchem et al. [54] found asthma prevalence 30% to 64% lower among youth who reported no BBB. Parchem et al. [55] found that BBB characterized transgender and gender diverse youth who were actively engaged in social activism, whereas hopelessness characterized youth who wanted to engage but had not done so. Parchem et al. [56] identified profiles characterized by high suicidality.

Intersecting identities were also associated with differences in access to protective factors and in their protective associations. Lessard et al. [4], drawing on the LGBTQ National Teen Survey, found that GSA presence was associated with lower levels of several forms of BBB, but not race- or ethnicity-based bullying. Burstein et al. [22] found that gender minority youth experienced BBB more often than cisgender peers and had less access to several protective factors, including financial stability, school or community membership, caring adults, and physical activity. Vigna et al. [16] found lower self-compassion among sexual and gender minority youth and weaker protection from self-compassion as BBB exposure increased. Among youth with comparable self-esteem and LGBTQ identity pride, Lawrence et al. [57], again drawing on the LGBTQ National Teen Survey, found 23% lower disordered eating among those without BBB exposure, indicating that these resources did not fully account for BBB-related differences in eating risk.

Evidence across the included studies indicated that cumulative targeting was associated with more severe and wide-ranging harm than targeting based on a single identity or characteristic. Multiply targeted youth reported lower school safety, poorer academic outcomes, greater fear and school avoidance, and higher suspension rates [31,38]. Greater cumulative BBB exposure was also associated with higher odds of nonsuicidal self-injury, suicidal ideation, forgone health care, sexual assault, and relationship abuse [8]. Cumulative targeting intensified the association between fear and school avoidance and was indirectly associated with avoidance through the negative effects of bullying [7,58]. Protective associations were also less consistent under cumulative exposure. School safety and social support were not consistently associated with better outcomes for multiply targeted youth, and the protective association of self-compassion diminished as BBB exposure increased [16,38]. Positive school climate was associated with lower baseline cumulative BBB victimization in the longitudinal study but did not predict changes over time [1]. Finally, BBB-related harm also extended to school systems. Baams et al. [59] estimated $276 million in annual California school funding losses associated with BBB and absences due to feeling unsafe.

Intersecting identities and identity-related resources also produced conditional and sometimes counterintuitive patterns. Price-Feeney et al. [60] found that victimization based on sexual orientation and gender expression was associated with depressive symptoms only among heterosexual and cisgender youth, despite higher exposure among sexual and gender minority youth. Wenzing et al. [61] found a more direct reversal. Strong ethnic identification intensified the association between discrimination and depressive symptoms, while strong religious identification intensified the association between discrimination and lower self-esteem. Disclosure and recognition of BBB also varied across identity and context, with immigrant girls most likely to tell no one and some rural adolescents minimizing racialized aggression as joking or denying its connection to racism [62,63].

#### 3.3.3. Proximal School and Peer Activation Processes

Whereas the first research question identified school and relational resources associated with protection, evidence across 22 studies indicated that proximal school and peer processes shaped whether youth could use those resources during and after BBB. School practices, supportive adults, peer relationships, and positive school climates did not protect youth simply because they were present. Studies linked their protective associations to whether students perceived school practices as fair, viewed adults as responsive and competent, and considered peer intervention credible and socially feasible.

Several studies identified school fairness and supportive practices as conditions associated with protection. Burgess et al. [2] found that perceptions of school fairness mediated the association between BBB and school belonging. Day et al. [33] associated supportive practices with lower homophobic bullying and greater school connectedness, whereas punitive practices were associated with neither outcome. Two analyses of the LGBTQ National Teen Survey by Lessard et al. [4,31] and Ioverno et al. [41] further found that reductions in BBB or perceived unsafety partly accounted for associations between inclusive school resources and health or school outcomes.

Adult responsiveness also shaped downstream outcomes. Joo et al. [58] found that supportive adults weakened the indirect association between cumulative targeting and school avoidance through the negative effects of bullying, although adult support did not eliminate this indirect association. Ramirez et al. [47] found that school personnel often understood general antibullying procedures but lacked the preparation, coordination, and BBB-specific guidance needed to recognize and respond to identity-based harm.

Peer relationships and intervention conditions also shaped whether protection became available in practice. Williams et al. [29] found that fear, peer relationships, and confidence in adults’ ability to respond influenced bystander behavior, while students rarely identified seeking adult assistance as a viable response. Gönültaş and Mulvey [27] found that theory of mind predicted greater help-seeking only when youth perceived positive peer norms, indicating that social-cognitive capacity did not independently translate into protective action. In a quasi-experimental evaluation, Zambuto et al. [43] found that an antibullying intervention was associated with reduced ethnic victimization only in classrooms where at least one peer educator had an immigrant background, indicating that the intervention’s effects depended on representation among peer educators. Longitudinal evidence complicated assumptions about relational protection. Ogg et al. [24] found that positive teacher–student and student–student relationships did not predict lower subsequent exposure to biased language. Instead, biased-language exposure predicted poorer later relationships, particularly among boys. Fusco et al. [64] showed a similar reversal of direction. Youth involved as BBB victims or bully-victims reported lower school social support, and the study modeled support as an outcome rather than a protective predictor.

Broader measures of school climate and support also produced conflicting findings. Using the National Crime Victimization Survey School Crime Supplement (NCVS-SCS), Mulvey et al. [38] found that social support was associated with fewer negative outcomes among youth experiencing general bullying but not among youth experiencing BBB. Using later waves from the same national survey program, Sahin-Ilkorkor [65] found the opposite pattern for social support, which showed a stronger buffering association among BBB victims than among non-BBB victims. Berkowitz et al. [5] found that moving from a less positive to a more positive school climate corresponded to a 32.6% lower predicted probability of sadness or hopelessness among nonvictimized youth but only a 1.7% lower probability among youth exposed to BBB. These findings indicated that proximal activation processes shaped whether available resources became credible, accessible, and usable forms of protection.

## 4. Discussion

This narrative synthesis produced two central conclusions. First, protective factors operated across individual, interpersonal, and institutional levels, but relational and institutional resources were more frequently associated with protection than individual strengths alone. This pattern reflects how often supportive findings appeared across studies rather than the relative strength of the evidence for each type of resource. Self-compassion, identity pride, family cohesion, supportive relationships, GSAs, caring school personnel, positive school climate, and inclusive practices all showed protective associations, yet no single factor was consistently associated with reduced BBB exposure or harm across youth, identities, and outcomes. Second, protection varied according to macro context, intersecting identities and cumulative targeting, and proximal school and peer processes. Protective factors were therefore not static resources with uniform effects. Their value appeared to depend on the broader environment, the nature and extent of targeting, and whether youth could access and use support when BBB occurred.

Viewed through a healthcare lens, these findings position BBB as both a preventable exposure and a contextual factor relevant to assessment, help-seeking, and recovery. The distinction between reducing exposure and reducing harm is especially important for healthcare professionals, who may encounter youth after victimization has already occurred. Individual coping resources may support recovery, but sustained protection may depend on whether families, schools, healthcare providers, and broader systems can recognize the exposure and coordinate an effective response.

Together, these findings suggest that protection from BBB emerges through interactions among resources distributed across ecological systems. EST helps locate these resources within relationships, schools, communities, and broader sociopolitical environments [9]. COR theory may help explain why BBB can produce widespread harm and why isolated supports often prove insufficient. From a COR perspective, stress occurs when valued resources are threatened, lost, or inadequately restored [10]. BBB can threaten identity affirmation, belonging, safety, trusted relationships, institutional confidence, and school engagement simultaneously. EST and COR therefore offer complementary frameworks for interpreting how BBB may produce harm and how protection may develop, accumulate, or fail across systems.

### 4.1. BBB as an Ecological Resource Threat

The findings position BBB as more than an interpersonal event associated with emotional distress. From a COR perspective, BBB may threaten resources across individual, relational, institutional, and social contexts. A single incident may undermine self-worth while also signaling that peers will not intervene, adults may not respond effectively, and the school does not fully recognize or protect the targeted identity. The effects of BBB may consequently extend beyond anxiety or depression to reduced belonging, avoidance, absenteeism, academic disengagement, and diminished trust in school institutions [2,7,21,59].

Intersectionality and cumulative targeting appeared to intensify these resource threats. Youth who held multiple marginalized social positions did not experience risk as the simple addition of separate identities. Rather, harm concentrated within particular combinations of identity, context, and exposure [49,50,51,52]. Youth targeted across multiple identities also experienced broader academic, psychological, and behavioral consequences than youth targeted on the basis of one identity or characteristic [8,31,38]. These findings suggest that cumulative targeting may threaten several resources at once while reducing the likelihood that any one support will address the full experience of harm. A GSA may affirm sexual or gender identity without addressing racial, religious, disability-related, or weight-based targeting. Similarly, self-esteem, identity pride, and self-compassion may support coping without eliminating the consequences of continued exposure [16,57]. Efforts to protect youth may need to account for the interaction between youths’ identities, the forms of BBB they experience, and the resources available across settings.

Findings from this narrative review also caution against treating marginalized groups as internally uniform. Identity-related resources did not operate consistently across all youth or outcomes. In some studies, identity identification or identity-related strengths offered limited protection or intensified associations between discrimination and distress under specific conditions [60,61]. These patterns do not imply that identity affirmation is harmful. Instead, they suggest that the protective value of identity-related resources may depend on the social environment and the type of threat youth encounter.

### 4.2. Protective Resources Across Systems

COR theory offers a useful explanation for how resources may accumulate across ecological levels. Resources can support the acquisition and preservation of additional resources, forming what COR theory describes as resource caravans [66]. The reviewed evidence was consistent with this principle. Individual resources appeared most useful when families, peers, adults, and institutions also affirmed youth identities and supported coping. Family cohesion, school belonging, peer support, and caring adults complemented psychological strengths by providing relational security and access to assistance [21,25].

Institutional resources may also combine several forms of protection. GSAs, for example, can provide peer affiliation, identity affirmation, adult access, institutional visibility, and collective support. Reduced BBB exposure partly explained their associations with better mental health and school adjustment [4,31]. Inclusive curricula, trained personnel, and representative school practices may similarly reinforce one another by increasing institutional competence and communicating that schools recognize and protect marginalized identities [40,41]. Importantly, the available studies did not test complete resource caravans across individual, family, peer, school, community, and policy levels. The synthesis is consistent with the idea that aligned resources may offer stronger protection than isolated supports, which future research should test directly.

Findings of this review are also consistent with the COR concept of resource loss. Ogg et al. [24] found that biased-language exposure predicted poorer subsequent teacher and peer relationships rather than relationships predicting lower exposure. Fusco et al. [64] similarly found lower school social support among youth involved in BBB. BBB may therefore erode the same relationships and supports that other studies identify as protective. Once victimization weakens trust, belonging, or social connection, youth may have fewer resources available to manage future exposure. This loss process may help explain why individual strengths and general school climate provided weaker protection as BBB exposure increased [5,16].

### 4.3. Protection May Depend on Activation Conditions

Results of this review indicated that the presence of a resource did not guarantee that the resource functioned as protection. Youth needed to recognize, access, trust, and use resources during and after BBB. This distinction helps explain why similar supports showed stronger associations in some contexts and weak or null associations in others.

Fairness and responsiveness represented important activation conditions. School fairness partly explained the association between BBB and belonging, while supportive practices showed stronger associations with connectedness and lower homophobic bullying than punitive approaches [2,33]. Adult support also weakened the indirect association between cumulative targeting and school avoidance, although it did not eliminate that association [58]. These findings suggest that adults and procedures become protective when youth perceive them as equitable, competent, and responsive.

Ramirez et al. [47] illustrated why formal resources may fail. School personnel often understood general antibullying requirements but lacked the preparation and BBB-specific guidance needed to recognize and address identity-based harm. A reporting procedure may therefore exist without functioning as meaningful protection if youth doubt that adults will understand or respond effectively.

Peer intervention required similar conditions. Fear, anticipated social consequences, peer relationships, and confidence in adults influenced whether students defended targeted youth [29]. Social-cognitive skills predicted help-seeking only when youth perceived supportive peer norms [27]. Peer-led prevention was also associated with reduced ethnic victimization only when peer educators included youth with immigrant backgrounds, suggesting that representation influenced intervention credibility [43].

These findings distinguish available resources from activated resources. A peer may possess empathy but remain silent when intervention threatens social standing. A student may know that supportive adults exist but avoid disclosure when previous responses appear ineffective. A school may adopt an inclusive policy but fail to train staff or establish credible procedures. Thus, protection may depend not only on which resources exist, but also on whether immediate social and institutional conditions make their use feasible.

### 4.4. Macro Conditions and the Resource Environment

Results of this review indicated that macro conditions were associated with BBB risk and with the environments in which school resources operated. Political events, litigation, laws, religious ideology, and community norms communicated which identities institutions recognized and whether schools had the authority, capacity, or motivation to respond. Broad legal indicators showed weaker associations than teacher training, inclusive curricula, and enforceable remedies [1,40]. Enforceable remedies were also associated with reduced homophobic bullying in a quasi-experimental analysis of litigation that produced a remedy favorable to students [44].

Political and social climates also influenced school-level risk. Homophobic bullying increased during the period surrounding California’s Proposition 8 vote, although schools with GSAs experienced smaller increases [32]. Other political effects varied by BBB type, school level, local context, and time period [45]. National inclusivity also mattered through its interaction with school outness, safety, and peer support rather than through a uniform direct effect [36].

Taken together, macro conditions may function as resource passageways that support or obstruct protection [67]. Legal recognition, enforceable procedures, and inclusive public norms can strengthen school resources. In contrast, hostile political discourse, exclusionary ideology, or vague mandates may weaken institutional legitimacy and leave students uncertain about whether schools will recognize their experiences [23,46,47].

### 4.5. Implications for Healthcare Practice, Schools, and Policy

For pediatric, primary care, behavioral health, and school-based healthcare professionals, BBB should be considered when youth present with emotional distress, self-harm risk, disordered eating, sleep problems, school avoidance, or unexplained disengagement. Assessment can include whether distress is connected to identity-based victimization, whether targeting is cumulative or ongoing, whether the youth feel safe disclosing their experience, and whether available adult or institutional responses are trusted. When BBB is identified, providers can assess immediate safety, support recovery, and, with appropriate consent, coordinate with caregivers and school personnel to reduce ongoing exposure and restore safety and connection. Because the included studies did not evaluate clinical screening tools or healthcare interventions, these recommendations reflect clinical implications of the evidence rather than tested protocols.

Importantly, the findings argue against approaches that place primary responsibility on targeted youth. Psychological strengths may support coping, but they cannot substitute for safe relationships, responsive adults, inclusive school structures, and enforceable institutional practices. Schools should therefore pair individual support with coordinated interventions across relational and institutional levels. Personnel training should address BBB as a distinct form of identity-based harm rather than treating it solely as general bullying. Staff need preparation to recognize cumulative targeting, respond to intersecting identities, and distinguish prevention of future exposure from support following victimization. Schools should also assess whether students view reporting procedures as accessible, confidential, credible, and likely to produce meaningful action.

Institutional resources should reflect the full range of student identities and BBB experiences. GSAs, inclusive curricula, affirming personnel, and identity-responsive programs showed protective associations, but no resource addressed every form of targeting. Schools should examine which students use available supports, which forms of BBB those supports address, and which groups remain underserved. Peer interventions should address social risk, peer norms, and confidence in adult follow-through rather than relying solely on empathy or willingness to help. Meaningful representation among peer leaders may strengthen credibility, but schools must pair peer-based programs with responsive adult systems so that students do not bear primary responsibility for preventing BBB. Likewise, policy should emphasize implementation and accountability. Enumerated protections may communicate institutional commitment, but effective protection also requires training, procedures, staffing, inclusive curricula, and ongoing evaluation of students’ experiences.

### 4.6. Limitations

This review synthesized 59 empirical studies representing multiple identities, BBB forms, methodological approaches, and ecological levels. The full-text coding process distinguished reduced exposure from reduced harm and retained null, conditional, and contrary findings. Integrating EST and COR theory also supported interpretation across individual, relational, institutional, and macro contexts. However, the evidence base relied heavily on cross-sectional studies, limiting causal and temporal conclusions. The MMAT appraisal identified recurring limitations. Of the 51 nonrandomized studies, 15 did not adequately account for confounding and 13 did not report sufficient information to assess completeness of outcome data. Neither randomized trial reported its randomization procedure in enough detail to judge, and one reported baseline differences between groups. Quality ratings also could not offset the predominance of cross-sectional designs. Studies varied in their definitions and measures of BBB, protective factors, cumulative targeting, school climate, and outcomes. Narrative synthesis also requires interpretive judgment when organizing heterogeneous evidence. Although structured full-text coding supported consistency, other reviewers might group findings differently. Screening and full-text assessment were conducted jointly by two reviewers rather than independently, so inter-rater agreement was quantified only for primary factor coding. The approach identified patterns and contradictions but could not estimate pooled effects or determine which protective factors were most effective. In addition, several publications drew from the same or related datasets, which reduced the independence of the evidence base and may have given disproportionate weight to findings reported across multiple articles. CINAHL and Embase were not searched. Although their relevant coverage overlapped with PubMed and Web of Science in preliminary screening, some nursing and allied health literature may have been missed. The supplementary searches of Google Scholar and the Mason Catalog and More discovery search were screened in order of relevance without recording the number of results screened, which limits the reproducibility of this part of the search. The review also followed an a priori protocol that was not registered.

Research represented sexual orientation, gender identity, race, and ethnicity more extensively than religion, disability, immigration status, or body size. This uneven representation limits the generalizability of the proposed framework because the protective resources and proposed mechanisms identified here may work differently for less-studied identities and forms of BBB. School-based surveys may also underrepresent youth who disengaged from school or experienced severe exclusion. Finally, the analytic categories overlapped by design. They represent complementary dimensions of protection rather than independent causal processes.

### 4.7. Implications for Future Research

Future studies should distinguish resources that reduce BBB exposure from those that reduce harm after exposure. These processes require different designs, measures, and intervention targets. Longitudinal and experimental research is also needed to clarify directionality because BBB may erode relationships and support rather than merely occur in their absence. Researchers should test how resources combine across individual, family, peer, school, community, and policy systems. Such studies could examine whether aligned resources produce additive or interactive protection and whether the loss of one resource initiates wider resource-loss processes. Research on cumulative targeting should measure the number, frequency, severity, and combinations of targeted identities. Studies should also assess activation directly by measuring whether youth know about resources, trust them, use them, and experience them as effective. Finally, researchers should continue reporting null, contrary, and subgroup-specific findings, which are essential for identifying the limits of universal approaches.

## 5. Conclusions

The findings suggest that protection from BBB depends on more than strengthening individual youth or adopting isolated policies. BBB can threaten identity, belonging, safety, relationships, institutional trust, and school engagement. Protective resources may reduce the likelihood of exposure, lessen harm following victimization, and support recovery. For healthcare and other youth-serving professionals, this underscores the importance of recognizing BBB as a potential source of harm, identifying its effects on youth well-being and functioning, and supporting recovery when victimization occurs. Because most included studies were cross-sectional, these conclusions reflect associations rather than established causal or comparative effects.

Prevention and post-exposure protection both depend on systems that can recognize identity-based harm, reduce opportunities for targeting, respond effectively when BBB occurs, and sustain the resources youth need afterward. For schools and policymakers, this requires inclusive climates and practices, credible adult and peer responses, accessible relational supports, and policy conditions that reinforce accountability. Protecting youth ultimately requires environments that seek to prevent identity-based harm and respond in ways that preserve safety, connection, trust, and engagement when harm occurs.

## Figures and Tables

**Table 1:** Primary Factor Type Distribution (*n* = 59).

Primary Factor Type	Studies (*n*)	% of Final Sample
Protective factors	13	22.0%
Mechanisms	18	30.5%
Protective factors + Mechanisms	28	47.5%
Total	59	100%

**Note.** Primary Factor Type reflects each study’s overall analytic focus, coded once per study. “Protective factors + Mechanisms” indicates a study that both tested at least one protective factor and examined a proposed mechanism or contextual condition associated with how that factor operated.

**Table 2:** Cross-Tabulation of Primary Factor Type by Subcategory (*n* = 59 studies; 93 tags).

Subcategory	Protective Factors	Mechanisms	Protective Factors + Mechanisms	Row Total (Tags)	% of Studies (*n* = 59)
Institutional protective factors	6	0	17	23	39.0%
Interpersonal protective factors: Relatedness	2	0	5	7	11.9%
Interpersonal protective factors: Bystander	2	0	3	5	8.5%
Interpersonal protective factors: Family	1	0	1	2	3.4%
Individual: Self-awareness and self-regulation	0	0	3	3	5.1%
Individual: Prosocial behavior and competencies	2	0	0	2	3.4%
Mechanism 1: Macro climate and policy context	0	2	10	12	20.3%
Mechanism 2: Intersectionality and cumulative targeting	0	12	5	17	28.8%
Mechanism 3: Proximal activation processes	0	5	17	22	37.3%
Column Total	13	19	61	93	—

**Note.** Cell values are subcategory tags, not studies. Each study could carry up to three subcategory tags, with no more than one tag per subcategory. The final column is the percentage of the 59 studies carrying each tag, and percentages sum to more than 100%. Studies classified as Mechanisms carry only mechanism subcategory tags because they did not test a protective factor. Tag counts describe how often a category was coded, not the strength of evidence for it. Mechanism subcategories describe proposed or inferred pathways and contextual processes rather than causal processes established by the included studies.

## Data Availability

No new data were created or analyzed in this study. Data sharing is not applicable to this article.

## Supplementary Materials

The following supporting information can be downloaded at https://www.mdpi.com/article/10.3390/healthcare14213589/s1, Supplemental Data S1, full data extraction matrix; Supplemental Table S1, Characteristics of Included Studies; Supplemental Table S2, search strategies, sources, dates, and limits; Supplemental Table S3, methodological quality ratings of included studies (MMAT). References [1,2,3,4,5,7,8,9,12,13,14,16,17,18,19,20,21,22,23,24,25,26,27,28,29,30,31,32,33,34,35,36,37,38,39,40,41,42,43,44,45,46,47,48,49,50,51,52,53,54,55,56,57,58,59,60,61,62,63,64,65] are cited in the Supplementary Materials.

## Abbreviations

The following abbreviations are used in this manuscript:

BBB	Bias-based bullying
COR	Conservation of Resources theory
EST	Ecological Systems Theory
GSA	Gender–sexuality alliance
SGM	Sexual and gender minority
