# Supplementary material for: From Risk to Resilience: A Narrative Synthesis of Protective Factors and Proposed Mechanisms in Bias-Based Bullying Among Youth (2016–2026)
Source: Healthcare (Basel). 2026 Oct 23;14(21):3589. doi: 10.3390/healthcare14213589 (PMC13762580; doi:10.3390/healthcare14213589)
Supplement: Supplementary file 1 [file healthcare-14-03589-s001.zip › Supplemental Table S1.pdf]

Supplemental Table S1. **Characteristics of Included Studies (n = 58)**

Study	Year	Design	Sample (N)	Data Source	Primary Factor Type
António et al.	2024	Experimental	170	Primary experimental data: 170 adolescents, Lisbon secondary schools	Protective factors
Baams et al.	2017	Cross-sectional	784,280	California Healthy Kids Survey, 2011–2013	Protective factors
Berkowitz et al.	2025	Cross-sectional	1,025,876	California Healthy Kids Survey, 2017–2019	Protective factors + Mechanisms
Burgess et al.	2023	Cross-sectional	2,600	Primary data: one rural Northeastern school district, grades 6–12 (No Place for Hate project)	Protective factors + Mechanisms
Burstein et al.	2024	Repeated cross-sectional	16,288; 10,792 (2 waves)	Dane County Youth Assessment 2018 and 2021, Wisconsin	Protective factors + Mechanisms
Day et al.	2016	Cross-sectional	337,945 students; 62,448 teachers	California Healthy Kids Survey + California School Climate Survey (teachers), 745 high schools	Protective factors + Mechanisms
Eisenberg et al.	2022	Cross-sectional	64,510 students (216 schools)	Minnesota Student Survey 2019 + 2018 CDC School Health Profiles	Protective factors
Eisenberg et al.	2024	Cross-sectional	80,456	Minnesota Student Survey 2019	Mechanisms
Fish et al.	2023	Cross-sectional	728,204	California Healthy Kids Survey, 2013–2015	Mechanisms
Fu et al.	2023	Cross-sectional	8,977 (of 69,244)	Maryland Safe and Supportive Schools (MDS3) survey 2015	Protective factors
Fusco et al.	2025	Cross-sectional	639	AmeriSpeak Teen Panel / Peer Experiences Project (2021 survey wave)	Mechanisms
Galán et al.	2021	Cross-sectional	3,939	Primary data: high school survey, Pittsburgh, PA, October 2018	Mechanisms
Gill & Govier	2023	Repeated cross-sectional	2,817,487	California Healthy Kids Survey, 2013–2019 (pooled waves)	Protective factors + Mechanisms
Gönültaş & Mulvey	2021	Cross-sectional	179	Primary data: 179 sixth and ninth graders, single U.S. school district	Protective factors
Gower et al.	2021	Cross-sectional	2,404	Minnesota Student Survey 2013 + 2014 CDC School Health Profiles	Protective factors + Mechanisms
Gower et al.	2022	Cross-sectional	124,778	Minnesota Student Survey 2019	Mechanisms
Gower et al.	2023	Cross-sectional	80,456; 512,067 (2 datasets)	Minnesota Student Survey 2019 + California Healthy Kids Survey 2017–2019	Mechanisms
Gower et al.	2025	Cross-sectional	80,456; 512,067 (2 datasets)	Minnesota Student Survey 2019 + California Healthy Kids Survey 2017–2019	Protective factors + Mechanisms
Haines-Saah et al.	2018	Qualitative (cross-sectional)	27	Primary qualitative data: 27 adolescents, rural northern British Columbia (RADAR project, 2012–2014)	Mechanisms
Hatzenbuehler et al.	2019	Quasi-experimental	4,977,557 (student-level)	California Healthy Kids Survey, 2001–2014 (14 waves)	Protective factors + Mechanisms
Hatzenbuehler et al.	2022	Quasi-experimental	1,448,778	California Healthy Kids Survey, 2001–2016 (15 waves)	Protective factors + Mechanisms

Study	Year	Design	Sample (N)	Data Source	Primary Factor Type
Hitti et al.	2023	Cross-sectional	168	Primary data: 168 ninth graders, two public high schools, one Southeastern U.S. district	Protective factors + Mechanisms
Holt et al.	2025	Cross-sectional	7,402	Primary data: 11 high schools, Northeastern U.S., fall 2019	Protective factors
Ioverno	2023	Cross-sectional	66,851	EU-LGBTI II Survey 2019 (EU Fundamental Rights Agency)	Protective factors + Mechanisms
Ioverno et al.	2025	Cross-sectional	17,733	Online SGM school survey, September 2020–January 2022	Protective factors + Mechanisms
Joo, Kim, et al.	2023	Cross-sectional	1,177	NCVS School Crime Supplement 2017	Mechanisms
Joo, Lee, et al.	2023	Repeated cross-sectional	629–1,197 (3 waves)	NCVS School Crime Supplement 2015, 2017, 2019	Protective factors + Mechanisms
Kidwai & Smith	2024	Cross-sectional	200 schools	Primary data: 200 school anti-bullying policies (United Against Bullying programme); pupil survey data from 131 schools	Protective factors
Kumaria et al.	2023	Cross-sectional	7,291	Healthy Minds Study 2018–2019 (college students)	Protective factors + Mechanisms
Lawrence et al.	2023	Cross-sectional	14,968	Minnesota Student Survey 2019 (sexually active 9th/11th graders)	Mechanisms
Lawrence et al.	2024	Cross-sectional	11,083	LGBTQ National Teen Survey 2017	Protective factors + Mechanisms
Lessard, Puhl, et al.	2020	Cross-sectional	17,112	LGBTQ National Teen Survey 2017	Protective factors + Mechanisms
Lessard, Watson, et al.	2020	Cross-sectional	17,112	LGBTQ National Teen Survey 2017	Protective factors + Mechanisms
McCauley et al.	2025	Cross-sectional	13,500	2022 National LGBTQ Teen Study	Protective factors
Meng et al.	2025	Cross-sectional	≈458,963	California Healthy Kids Survey, 2017–2019	Mechanisms
Miller et al.	2022	Cross-sectional	481	Primary data: 481 middle school youth, 6 public middle schools, Northeastern U.S.	Mechanisms
Moran et al.	2020	Quasi-experimental	146	Primary data: sixth graders in one low-income, ethnically blended middle school (agricultural community)	Protective factors
Mulvey et al.	2018	Cross-sectional	678	NCVS School Crime Supplement 2015	Protective factors + Mechanisms
Newman et al.	2018	Qualitative (cross-sectional)	16 key informants	Primary qualitative data: 16 key informants (service providers, educators), Greater Toronto Area	Mechanisms
O'Malley et al.	2022	Cross-sectional	74,501	California Healthy Kids Survey (Core), 381 high schools	Protective factors + Mechanisms
Ogg et al.	2021	Longitudinal (panel)	501	Primary data: one suburban Midwestern middle school; 4 waves, 2016–2018	Protective factors + Mechanisms
Parchem et al.	2024	Cross-sectional	4,566	Dane County Youth Assessment 2021, Wisconsin (LGBTQ+ subsample)	Mechanisms
Parchem, Brightly-Brown, et al.	2025	Cross-sectional	698	Dane County Youth Assessment 2021, Wisconsin (transgender and gender diverse students)	Mechanisms
Parchem, Gower, et al.	2025	Cross-sectional	90,367	Minnesota Student Survey 2022	Mechanisms
Parchem, Rudo-Stern, et al.	2025	Cross-sectional	17,794	Dane County Youth Assessment 2021, Wisconsin	Protective factors

Study	Year	Design	Sample (N)	Data Source	Primary Factor Type
Parodi et al.	2026	Longitudinal (panel)	639	AmeriSpeak Teen Panel / Peer Experiences Project (nationally representative; 4 waves)	Protective factors + Mechanisms
Price-Feeney et al.	2018	Cross-sectional	5,542	Teen Health and Technology Study, national online survey 2010–2011	Mechanisms
Ramirez et al.	2023	Qualitative (cross-sectional)	19 educators (7 teams)	Primary qualitative data: 7 school teams (19 educators), Minnesota	Protective factors + Mechanisms
Sahin-Ilkorkor	2025	Repeated cross-sectional	NR (NCVS-SCS, repeated waves)	NCVS School Crime Supplement, 2015–2022	Mechanisms
Shah et al.	2021	Cross-sectional	481	Primary data: 6 public middle schools, Northeastern U.S.	Protective factors + Mechanisms
Shank & Troshynski	2024	Qualitative (cross-sectional)	176 (+15, town halls)	Secondary qualitative data: community-meeting transcripts (2017–2018) and town halls (2019), one school district	Protective factors + Mechanisms
Sherwood et al.	2025	Cross-sectional	12,764	Multi-country SGM survey, 2020–2021	Protective factors + Mechanisms
Thomas et al.	2025	Cross-sectional	8,503	National retrospective survey of adults aged 16+ (telephone interviews)	Protective factors
Vigna et al.	2018	Cross-sectional	1,821	Dane County Youth Assessment (2 suburban high schools)	Protective factors + Mechanisms
Wenzing et al.	2024	Cross-sectional	105	Primary data: Identity Project, culturally diverse schools	Mechanisms
Williams et al.	2018	Qualitative (cross-sectional)	14	Primary qualitative data: 14 high school students	Protective factors + Mechanisms
Zambuto et al.	2022	Quasi-experimental	1,570	Primary quasi-experimental data: 74 classes, grades 7–10, Tuscany	Protective factors + Mechanisms
Zych & Llorent	2021	Cross-sectional	2,139	Primary data: 22 secondary schools, Andalusia	Protective factors

Note: Studies are ordered alphabetically by first author. Where two or more included studies share the same first author and publication year, the differing second author distinguishes the citation. NR = not reported as a single figure in the extracted sample description.
