# Supplementary material for: From Risk to Resilience: A Narrative Synthesis of Protective Factors and Proposed Mechanisms in Bias-Based Bullying Among Youth (2016–2026)
Source: Healthcare (Basel). 2026 Oct 23;14(21):3589. doi: 10.3390/healthcare14213589 (PMC13762580; doi:10.3390/healthcare14213589)
Supplement: Supplementary file 1 [file healthcare-14-03589-s001.zip › Supplemental Table S2.pdf]

Supplemental Table S2. Search strategies, sources, dates, and limits.

A. Search overview

Element	Description
Review type	Literature review with theory-informed narrative synthesis
Search period	May 4–July 29, 2026 (bibliographic databases and supplementary sources)<br>PubMed search updated September 29, 2026
Preliminary scoping	Preliminary scoping searches, beginning in March 2026, informed database selection; the searches reported in this table were conducted May 4–July 29, 2026.
Bibliographic databases	APA PsycInfo and APA PsycArticles (EBSCOhost, searched together); ERIC (EBSCOhost); Web of Science (Clarivate); PubMed (National Library of Medicine)
Supplementary sources	Google Scholar; Mason Catalog and More discovery search (Primo; George Mason University Libraries); reference lists of included articles
Core concepts	(1) Bias-based or identity-based bullying and victimization; (2) protective, promotive, or contextual factors (e.g., resilience, school climate, belonging, connectedness, peer support, bystander behavior). Syntax was adapted for each database while maintaining conceptual consistency.
Search fields and settings	EBSCOhost: full-text searching enabled ("Also search within the full text of the articles"), allowing retrieval of articles in which BBB terminology appeared only in the body text. Web of Science: Topic (TS) field. PubMed: all fields.
Spelling variant	The term "biased-based bullying" was included intentionally to capture a common misspelling in indexed records.
Eligibility applied at screening	English-language, peer-reviewed empirical studies of youth published from 2016 through 2026 (Section 2.3). Grey literature was excluded.
Databases not searched	CINAHL and Embase; their coverage of journals relevant to school-based youth bullying was expected to overlap substantially with PubMed/MEDLINE and Web of Science.
Record management and deduplication	Records were managed in Zotero and a master Excel workbook; duplicates were reconciled using title, author, publication year, DOI, and full-text comparison.

B. Database-specific search strategies

Database	Platform	Search string(s)	Fields and settings	Records
APA PsycInfo and APA PsycArticles	EBSCOhost	"bias-based bullying" OR "biased-based bullying"	Full-text searching enabled; searched together; run with and without a longitudinal filter	642 (combined)
ERIC	EBSCOhost	(a) ((bully* OR victimization OR harassment OR cyberbullying) N5 (racism OR racial OR ethnic OR xenophobia OR homophobia OR transphobia OR "sexual minority" OR "gender minority" OR disability OR "religious discrimination" OR bias-based OR discriminatory)) AND (protect* OR buffer* OR resilience OR "protective factor*" OR moderator* OR mediator* OR "school climate" OR "teacher intervention*" OR "family school" OR connectedness OR belonging OR bystander* OR "peer support" OR identity OR "ethnic-racial identity")<br>(b) "bias-based bullying" AND (protective factors or resilience or promotive factors or buffer)<br>(c) "bias-based bullying" AND (protective factors or resilience or promotive factors or buffer) AND context<br>(d) ("bias-based bullying" OR "biased-based bullying")	—	131

Database	Platform	Search string(s)	Fields and settings	Records
Web of Science	Clarivate	TS=((("bias-based" OR "identity-based") NEAR/3 (bully* OR victimization OR harassment)) OR ((bully* OR victimization OR harassment) NEAR/3 (bias OR prejudice OR discrimination))) AND TS=(protect* OR resilience OR "school climate" OR connectedness OR belonging OR "peer support" OR bystander*)	Topic (TS) field	424
PubMed	National Library of Medicine	Original search (May 4–July 29, 2026): ("bias-based bullying" OR "bias-based victimization" OR "identity-based bullying" OR "identity-based victimization") AND (protect* OR resilience OR "school climate" OR connectedness OR belonging OR "peer support")<br>Updated search (September 29, 2026): same search string	All fields	11 (original search)<br>Updated search: 1 additional eligible study

C. Supplementary searching

Source	Search terms	Procedure	Records
Google Scholar	longitudinal bias-based bullying adolescent<br>bias-based bullying + "protective factors" + "mental health outcomes"<br>"longitudinal" + "bias-based bullying"	Results screened in order of relevance; number of results screened not recorded	2 retained
Mason Catalog and More (Primo), George Mason University Libraries	"longitudinal" + "bias-based bullying"	Results screened in order of relevance; number of results screened not recorded	2 retained
Reference lists of included articles	—	Manual review of reference lists	1 article

D. Records identified by source

Source	Records	Notes
APA PsycInfo and APA PsycArticles (EBSCOhost)	642	Combined count across both databases
ERIC	131	
Web of Science	424	
PubMed (original search)	11	
**Bibliographic databases, total**	**1,208**	Before deduplication
Google Scholar	2	Records retained
Mason Catalog and More (Primo)	2	Records retained
Reference lists of included articles	1	Added at full-text stage
Updated PubMed search (September 29, 2026)	1	Additional eligible study added at full-text stage

Note. Screening, eligibility assessment, and inclusion are summarized in Figure 2. Records are reported as retrieved, before deduplication.
