# Supplementary material for: From Risk to Resilience: A Narrative Synthesis of Protective Factors and Proposed Mechanisms in Bias-Based Bullying Among Youth (2016–2026)
Source: Healthcare (Basel). 2026 Oct 23;14(21):3589. doi: 10.3390/healthcare14213589 (PMC13762580; doi:10.3390/healthcare14213589)
Supplement: Supplementary file 1 [file healthcare-14-03589-s001.zip › Supplemental Table S3.pdf]

Supplemental Table S3. Methodological quality ratings of included studies using the Mixed Methods Appraisal Tool (MMAT, version 2018).

**Qualitative studies (n = 5)** Criteria: 1.1 Approach appropriate; 1.2 Data collection adequate; 1.3 Findings derived from data; 1.4 Interpretation supported; 1.5 Coherence										
**Ref.**	**Study**	**Design**	**S1**	**S2**	**1.1**	**1.2**	**1.3**	**1.4**	**1.5**	**Notes**
21	Shank & Troshynski (2024)	Qualitative (cross-sectional)	Y	Y	Y	Y	Y	Y	Y	—
27	Williams et al. (2018)	Qualitative (cross-sectional)	Y	Y	Y	Y	Y	Y	Y	—
44	Newman et al. (2018)	Qualitative (cross-sectional)	Y	Y	Y	Y	Y	Y	Y	—
45	Ramirez et al. (2023)	Qualitative (cross-sectional)	Y	Y	Y	Y	Y	Y	Y	—
60	Haines-Saah et al. (2018)	Qualitative (cross-sectional)	Y	Y	Y	Y	Y	Y	Y	—

**Quantitative randomized controlled trials (n = 1)** Criteria: 2.1 Randomization appropriate; 2.2 Groups comparable at baseline; 2.3 Complete outcome data; 2.4 Assessors blinded; 2.5 Adherence										
**Ref.**	**Study**	**Design**	**S1**	**S2**	**2.1**	**2.2**	**2.3**	**2.4**	**2.5**	**Notes**
24	António et al. (2024)	Experimental	Y	Y	**CT**	**CT**	Y	**CT**	Y	2.1 CT: 'randomly assigned' only; sequence generation and concealment not described by the authors. 2.2 CT: baseline comparison across conditions not reported by the authors. 2.4 CT: self-reported intentions; participants saw only their own scenario, but blinding to hypotheses not reported by the authors.

**Quantitative nonrandomized studies (n = 51)** Criteria: 3.1 Representative sample; 3.2 Appropriate measures; 3.3 Complete outcome data; 3.4 Confounders accounted for; 3.5 Exposure as intended										
**Ref.**	**Study**	**Design**	**S1**	**S2**	**3.1**	**3.2**	**3.3**	**3.4**	**3.5**	**Notes**
1	Parodi et al. (2026)	Longitudinal (panel)	Y	Y	Y	Y	Y	Y	Y	—
2	Burgess et al. (2023)	Cross-sectional	Y	Y	Y	Y	Y	**N**	Y	3.4 N: SEM mediation model with no covariates.
3	Eisenberg et al. (2022)	Cross-sectional	Y	Y	Y	Y	**CT**	Y	Y	3.3 CT: student-level missing data not reported by the authors.
4	Lessard et al. (2020)	Cross-sectional	Y	Y	Y	Y	Y	Y	Y	—
5	Berkowitz et al. (2025)	Cross-sectional	Y	Y	Y	Y	Y	Y	Y	—
7	Joo et al. (2023)	Cross-sectional	Y	Y	Y	Y	Y	Y	Y	—
8	Galán et al. (2021)	Cross-sectional	Y	Y	Y	Y	Y	Y	Y	—
12	Gower et al. (2022)	Cross-sectional	Y	Y	Y	Y	**CT**	Y	Y	3.3 CT: listwise deletion by analysis; amount missing not reported by the authors.
15	Vigna et al. (2018)	Cross-sectional	Y	Y	Y	Y	Y	Y	Y	—
16	O'Malley et al. (2022)	Cross-sectional	Y	Y	Y	Y	Y	Y	Y	—
17	Zych & Llorent (2021)	Cross-sectional	Y	Y	Y	Y	Y	Y	Y	—
18	Hitti et al. (2023)	Cross-sectional	Y	Y	Y	Y	Y	Y	Y	—
19	Holt et al. (2025)	Cross-sectional	Y	Y	Y	Y	Y	Y	Y	—
20	Burstein et al. (2024)	Repeated cross-sectional	Y	Y	Y	Y	Y	Y	Y	—
22	Ogg et al. (2021)	Longitudinal (panel)	Y	Y	Y	Y	Y	Y	Y	—
23	Shah et al. (2021)	Cross-sectional	Y	Y	Y	Y	Y	Y	Y	—

**Quantitative nonrandomized studies (n = 51)** Criteria: 3.1 Representative sample; 3.2 Appropriate measures; 3.3 Complete outcome data; 3.4 Confounders accounted for; 3.5 Exposure as intended										
**Ref.**	**Study**	**Design**	**S1**	**S2**	**3.1**	**3.2**	**3.3**	**3.4**	**3.5**	**Notes**
25	Gönültaş & Mulvey (2021)	Cross-sectional	Y	Y	**CT**	Y	Y	Y	Y	3.1 CT: 179 low-income students from 3 schools in one Southeastern U.S. district; participation rate not reported by the authors.
26	Kumaria et al. (2023)	Cross-sectional	Y	Y	Y	Y	Y	Y	Y	—
28	Gower et al. (2021)	Cross-sectional	Y	Y	Y	Y	Y	Y	**CT**	3.5 CT: GSA presence (2014) and community resources (2018-19) measured after 2013 student reports; GSA status at the time of the 2013 survey not reported by the authors.
29	Lessard et al. (2020)	Cross-sectional	Y	Y	Y	Y	Y	Y	Y	—
30	Hatzenbuehler et al. (2019)	Quasi-experimental	Y	Y	Y	Y	Y	Y	Y	—
31	Day et al. (2016)	Cross-sectional	Y	Y	Y	Y	**CT**	Y	Y	3.3 CT: missing data not reported by the authors.
32	McCauley et al. (2025)	Cross-sectional	Y	Y	Y	Y	Y	Y	Y	—
33	Gower et al. (2025)	Cross-sectional	Y	Y	Y	Y	Y	Y	Y	3.4 Y: decision-tree (CHAID) analysis; demographic variables modeled jointly (see Section 2.5).
34	Sherwood et al. (2025)	Cross-sectional	Y	Y	Y	Y	Y	Y	Y	—
35	Fu et al. (2023)	Cross-sectional	Y	Y	Y	Y	Y	Y	Y	—
36	Mulvey et al. (2018)	Cross-sectional	Y	Y	Y	Y	Y	Y	Y	—
37	Kidwai & Smith (2024)	Cross-sectional	Y	Y	Y	Y	Y	**N**	Y	3.4 N: bivariate correlations only.
38	Ioverno (2023)	Cross-sectional	Y	Y	Y	Y	**CT**	Y	Y	3.3 CT: missing data not reported by the authors.
39	Ioverno et al. (2025)	Cross-sectional	Y	Y	Y	Y	Y	Y	Y	—
40	Moran et al. (2020)	Quasi-experimental	Y	Y	Y	Y	Y	**N**	**CT**	3.4 N: no comparison group; change over time not separated from other influences. 3.5 CT: implementation fidelity not reported by the authors.
41	Zambuto et al. (2022)	Quasi-experimental	Y	Y	**CT**	Y	**CT**	Y	**CT**	3.1 CT: 74 classes in 14 Tuscan schools; school recruitment and participation not reported by the authors. 3.3 CT: attrition not reported by the authors. 3.5 CT: implementation fidelity not reported by the authors.
42	Hatzenbuehler et al. (2022)	Quasi-experimental	Y	Y	Y	Y	Y	Y	Y	—
43	Gill & Govier (2023)	Repeated cross-sectional	Y	Y	Y	Y	Y	Y	Y	—
46	Fish et al. (2023)	Cross-sectional	Y	Y	Y	Y	Y	Y	Y	—
47	Eisenberg et al. (2024)	Cross-sectional	Y	Y	Y	Y	Y	Y	Y	3.4 Y: decision-tree (CHAID) analysis; demographic variables modeled jointly (see Section 2.5).
48	Lawrence et al. (2023)	Cross-sectional	Y	Y	Y	Y	**CT**	Y	Y	3.3 CT: missing data handling not reported by the authors beyond recoding unclear responses to missing. 3.4 Y: decision-tree (CHAID) analysis; demographic variables modeled jointly (see Section 2.5).
49	Meng et al. (2025)	Cross-sectional	Y	Y	Y	Y	**CT**	Y	Y	3.3 CT: restricted to non-missing suicidal ideation responses; number excluded not reported by the authors. 3.4 Y: decision-tree (CHAID) analysis; demographic variables modeled jointly (see Section 2.5).
50	Gower et al. (2023)	Cross-sectional	Y	Y	Y	Y	Y	Y	Y	3.4 Y: decision-tree (CHAID) analysis; demographic variables modeled jointly (see Section 2.5).
51	Parchem et al. (2024)	Cross-sectional	Y	Y	Y	Y	**CT**	Y	Y	3.3 CT: missing data not reported by the authors. 3.4 Y: latent class regression; demographic variables modeled jointly (see Section 2.5).
52	Parchem et al. (2025)	Cross-sectional	Y	Y	Y	Y	**CT**	Y	Y	3.3 CT: restricted to students with asthma data; number excluded not reported by the authors. 3.4 Y: decision-tree (CHAID) analysis; demographic variables modeled jointly (see Section 2.5).
53	Parchem et al. (2025)	Cross-sectional	Y	Y	Y	Y	**CT**	Y	Y	3.3 CT: missing data not reported by the authors. 3.4 Y: decision-tree (CHAID) analysis; demographic variables modeled jointly (see Section 2.5).
54	Parchem et al. (2025)	Cross-sectional	Y	Y	Y	Y	Y	Y	Y	3.4 Y: decision-tree (CHAID) analysis; demographic variables modeled jointly (see Section 2.5).
55	Lawrence et al. (2024)	Cross-sectional	Y	Y	Y	Y	**CT**	Y	Y	3.3 CT: analytic n = 11,083 of 17,112 survey participants; basis for the subsample not clearly reported by the authors. 3.4 Y: decision-tree (CHAID) analysis; demographic variables modeled jointly (see Section 2.5).

**Quantitative nonrandomized studies (n = 51)** Criteria: 3.1 Representative sample; 3.2 Appropriate measures; 3.3 Complete outcome data; 3.4 Confounders accounted for; 3.5 Exposure as intended										
**Ref.**	**Study**	**Design**	**S1**	**S2**	**3.1**	**3.2**	**3.3**	**3.4**	**3.5**	**Notes**
56	Joo et al. (2023)	Repeated cross-sectional	Y	Y	Y	Y	Y	**N**	Y	3.4 N: analytic plan (stepwise regression, PROCESS Model 14) describes no demographic covariates.
57	Baams et al. (2017)	Cross-sectional	Y	Y	Y	Y	Y	**N**	Y	3.4 N: survey-adjusted logistic regressions of absence on BBB types with no covariates described.
58	Price-Feeney et al. (2018)	Cross-sectional	Y	Y	Y	Y	Y	Y	Y	—
59	Wenzing et al. (2024)	Cross-sectional	Y	Y	Y	Y	Y	Y	Y	—
61	Miller et al. (2022)	Cross-sectional	Y	Y	Y	Y	**CT**	Y	Y	3.3 CT: missingness examined (e.g., 4.6% gender, 22% on a family cohesion item) but handling not clearly reported by the authors.
62	Fusco et al. (2025)	Cross-sectional	Y	Y	Y	Y	Y	Y	Y	—
63	Sahin-Ilkorkor (2025)	Repeated cross-sectional	Y	Y	Y	Y	**CT**	Y	Y	3.3 CT: restricted to victims with complete data on all variables; number excluded not reported by the authors.

**Quantitative descriptive studies (n = 1)** Criteria: 4.1 Sampling strategy relevant; 4.2 Representative sample; 4.3 Appropriate measures; 4.4 Low nonresponse bias; 4.5 Appropriate analysis										
**Ref.**	**Study**	**Design**	**S1**	**S2**	**4.1**	**4.2**	**4.3**	**4.4**	**4.5**	**Notes**
13	Thomas et al. (2025)	Cross-sectional	Y	Y	Y	Y	Y	Y	Y	—

Note. Y = yes; N = no; CT = can't tell (the information needed to rate the criterion was not reported, or was reported unclearly, by the study authors). S1 = clear research questions; S2 = collected data allow the research questions to be addressed. Ratings are presented by criterion; no overall score was calculated. Decision rules applied uniformly across studies: representativeness (3.1, 4.2) was rated as met when the target population and sample were clearly described, including population-based, census-type, program-wide, and large national or multinational samples of a specific population; complete outcome data (2.3, 3.3) was rated as met when missing data were handled with multiple imputation or maximum-likelihood methods, at least 80% of the sample was analyzed, only routine data-cleaning exclusions were reported, or the primary outcome was complete; confounding (3.4) was rated as met when models included full or partial covariate adjustment, stratified by key groups, jointly modeled demographic and social-position variables (decision-tree or latent class analyses), or used a comparison-group design; appropriate measures (3.2, 4.3) was rated as met when established or validated scales or standard survey items were used.
